# A Case of Chronic Expanding Pericardial Hematoma Treated Surgically

**DOI:** 10.7759/cureus.81439

**Published:** 2025-03-29

**Authors:** Kazuki Mori, Satoshi Takebayashi, Masato Morita, Masazumi Kume, Yuichi Nakazono

**Affiliations:** 1 Department of Cardiovascular Surgery, National Hospital Organization (NHO) Beppu Medical Center, Beppu, JPN; 2 Department of Pathology, National Hospital Organization (NHO) Beppu Medical Center, Beppu, JPN

**Keywords:** chronic expanding hematoma, idiopathic, mediastinal tumor, pericarditis, pericardium

## Abstract

Chronic expanding hematoma (CEH) is a rare condition characterized by a slowly enlarging hematoma. While chronic expanding hematomas can occur following trauma or surgery, their idiopathic presentation is exceedingly rare. An expanding hematoma can silently lead to diastolic cardiac dysfunction. A 69-year-old woman without a history of thoracic surgery or chest trauma presented with an incidentally discovered pericardial mass measuring 90 × 60 × 45 mm. Imaging revealed a mass with a calcified capsule that compressed the right heart in the anterior mediastinum. Despite initial attempts at off-pump resection, the procedure was converted to on-pump resection because of cardiac injury caused by extensive adhesions. The excised specimen was encapsulated and filled with an old hematoma. Histopathological examination results confirmed the diagnosis of chronic expanding hematoma with no evidence of malignancy. Despite being asymptomatic, the mass was significantly compressing the heart, necessitating surgical resection for diagnostic and therapeutic purposes.

## Introduction

Chronic expanding hematoma (CEH) is a rare condition characterized by a slowly enlarging hematoma. Although the exact etiology is unclear, CEH is often triggered by trauma and surgery [1]. However, idiopathic cases are exceedingly rare. Furthermore, CEH rarely occurs in the pericardial space, and its presence may lead to cardiac compromise secondary to cardiac compression. This report presents a case of incidentally diagnosed CEH in the pericardium that was surgically treated.

## Case presentation

**Figure 1:**
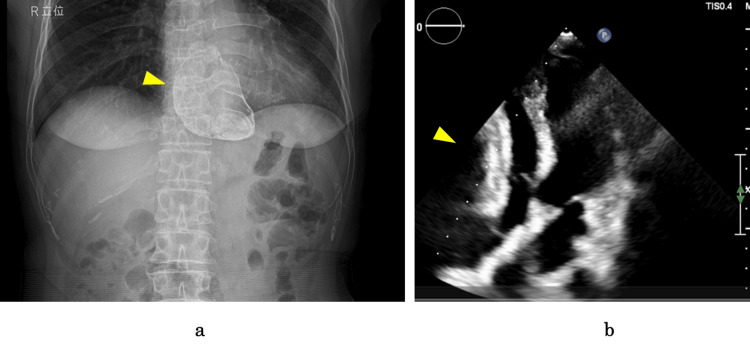
Preoperative X-ray image and echocardiography (a) X-ray image revealing a calcified mass within the cardiac shadow (arrow). (b) Transthoracic echocardiography demonstrating a mass anterior to the heart (arrow), resulting in right ventricular compression and diastolic dysfunction. This figure shows an apical four-chamber view.

A 69-year-old woman with a history of hypertension was incidentally found to have calcified mediastinal opacity on chest X-ray imaging (Figure 1a), which was performed for evaluation of her lower back pain. The patient was asymptomatic and had no history of thoracic surgery, chest trauma, or anticoagulant or antiplatelet use. The coagulation studies were unremarkable. There was no evidence of anemia or organ dysfunction, such as hepatic or renal impairment. The electrocardiogram showed no evidence of arrhythmia or ischemia. Echocardiography revealed a mass in the anterior cardiac region, causing compression of the right ventricle and right atrium (Figure 1b). A mild increase in velocity of 1.3 m/s was observed in the right ventricular outflow tract. The inferior vena cava was dilated to 19 mm, but respiratory variation was preserved.

**Figure 2:**
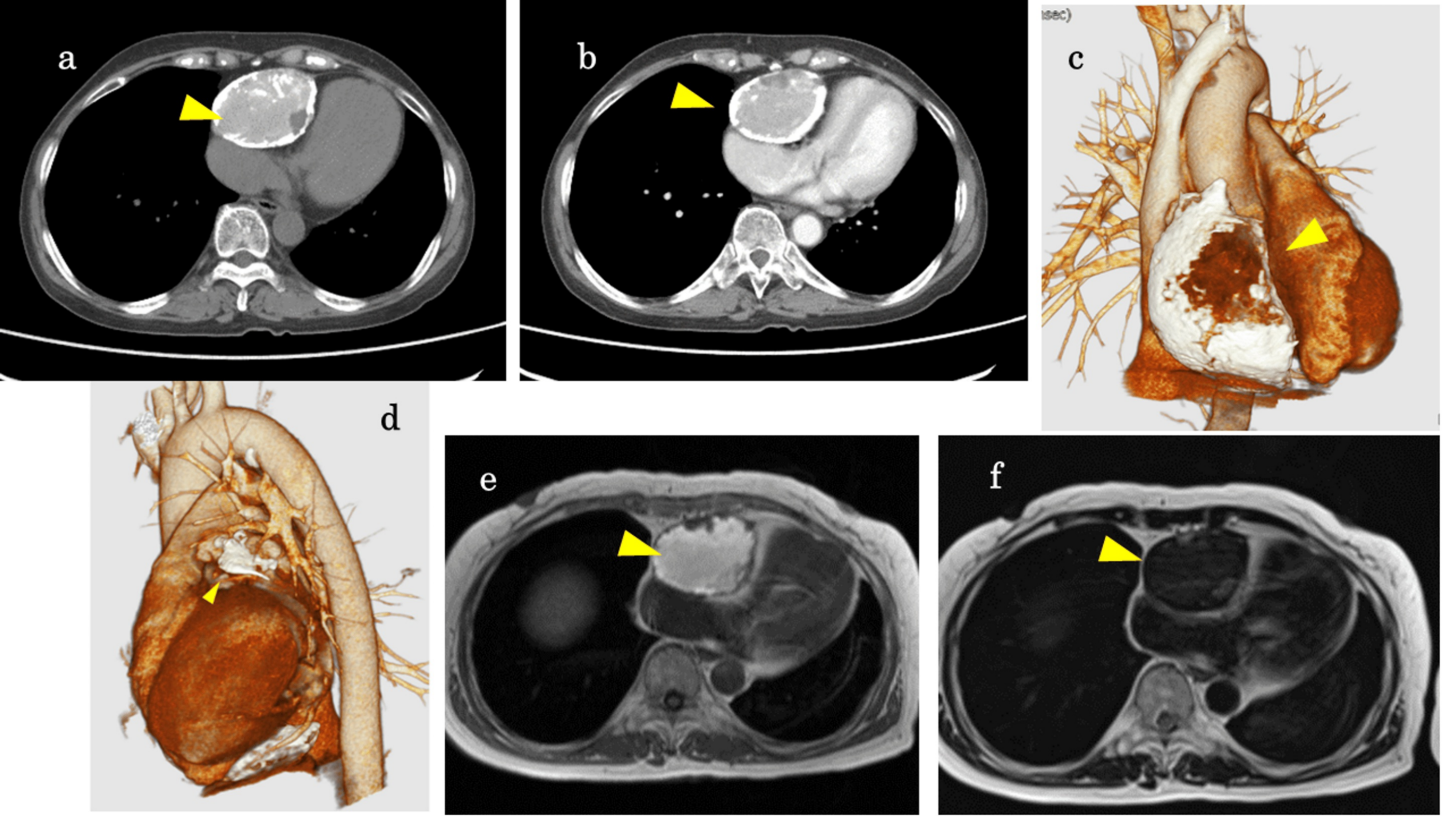
Preoperative computed tomography images and magnetic resonance images (a) Plain, (b) contrast-enhanced, and (c) 3D computed tomography images revealing a well-circumscribed 90 × 60 × 45 mm mass with a calcified capsule in the anterior mediastinum (arrow), causing extrinsic compression of the heart. Scattered calcifications were observed within the mass substance. (d) Calcific deposits were observed around the left atrial appendage, without evidence of a mass (arrow). (e) T1-weighted and (f) T2-weighted magnetic resonance images revealed a mass with predominantly high T1 and low T2 signal intensities (arrow). A mosaic pattern of the signal intensity was observed within the mass.

The internal structures of the lesion were obscured by posterior acoustic shadowing. Computed tomography (CT) revealed a mass measuring 90 × 60 × 45 mm in the anterior mediastinum (Figure 2a-2c). The mass had a calcified capsule and was significantly compressing the right heart. On CT, the mass showed high attenuation with some nodular components and calcification but no contrast enhancement. Calcification was also observed around the left atrial appendage; however, no mass lesion was identified in the same area (Figure 2d). No calcification was found in other parts of the pericardium. The lesion exhibited hyperintensity on T1-weighted and hypointensity on T2-weighted magnetic resonance images (MRI) (Figure 2e, 2f). The lesion's capsule exhibited hypointensity on both T1-weighted and T2-weighted images. The presence of a hematoma or a mediastinal tumor, including a teratoma or an ectopic thyroid tumor, was suspected. Consequently, a tumor resection was planned for diagnostic purposes.

**Figure 3:**
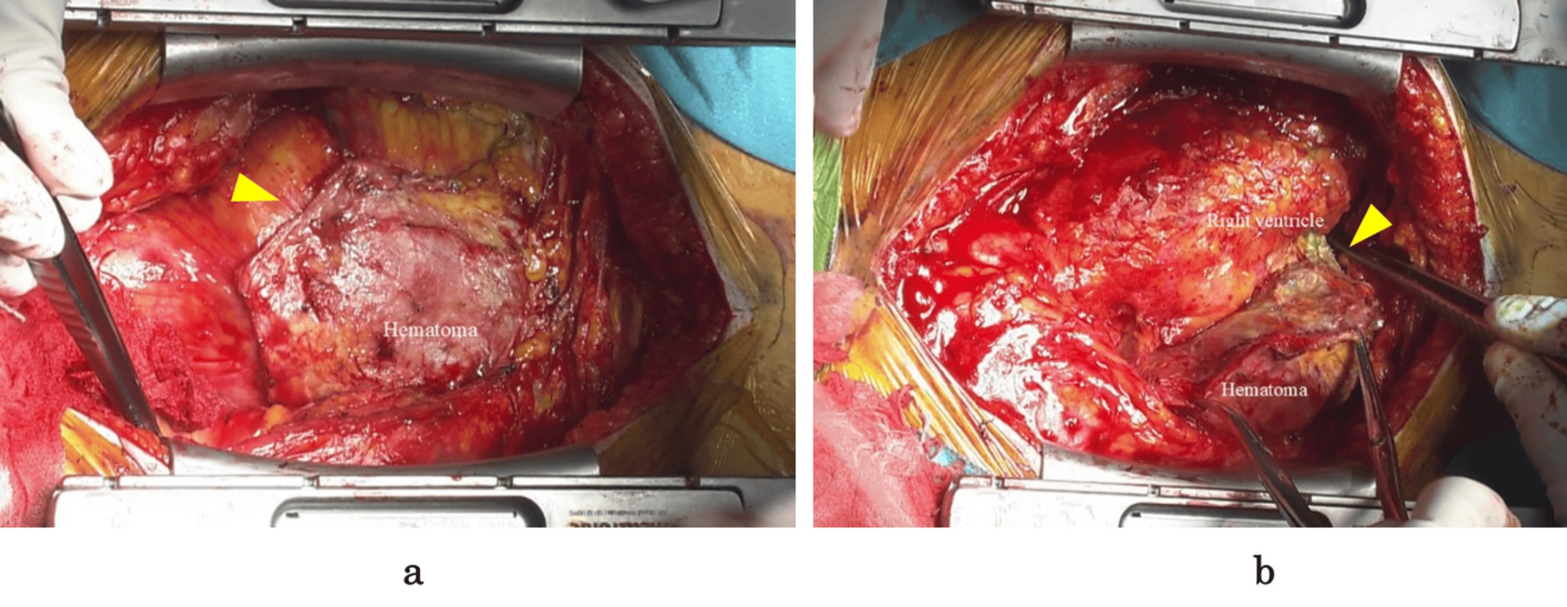
Intraoperative findings (a) A well-circumscribed mass (arrow) was found within the pericardial sac. (b) The lesion was firmly adherent to the heart (arrow). No epicardial thickening or calcification was noted.

A median sternotomy was performed. There was no evidence of adhesions posterior to the sternum. The pericardium was incised anterior to the ascending aorta, away from the lesion. No adhesion was observed between the aorta and the pericardium. However, adhesions were present within the pericardial sac, particularly between the lesion and the heart. Despite these adhesions, epicardial calcification or thickening was not noted (Figure 3a). The initial dissection focused on adhesion areas that were not directly related to the lesion. Subsequently, the adhesion between the tumor and the heart was carefully dissected using an ultrasonic scalpel. Owing to the dense adhesions surrounding the right atrial appendage, the dissection was gradually extended from the right ventricular periphery (Figure 3b). During en bloc resection of the lesion, the adhesion site to the right atrium was inadvertently damaged, which resulted in hemorrhage. For bleeding control, cardiopulmonary bypass was established through the cannulation of the left femoral artery and vein. After en bloc resection of the lesion with the adherent right atrial wall, the atrial defect was reinforced with a felt patch and closed with a continuous 4-0 polypropylene suture. Following weaning from cardiopulmonary bypass and placement of drainage tubes, the chest was sutured closed.

**Figure 4:**
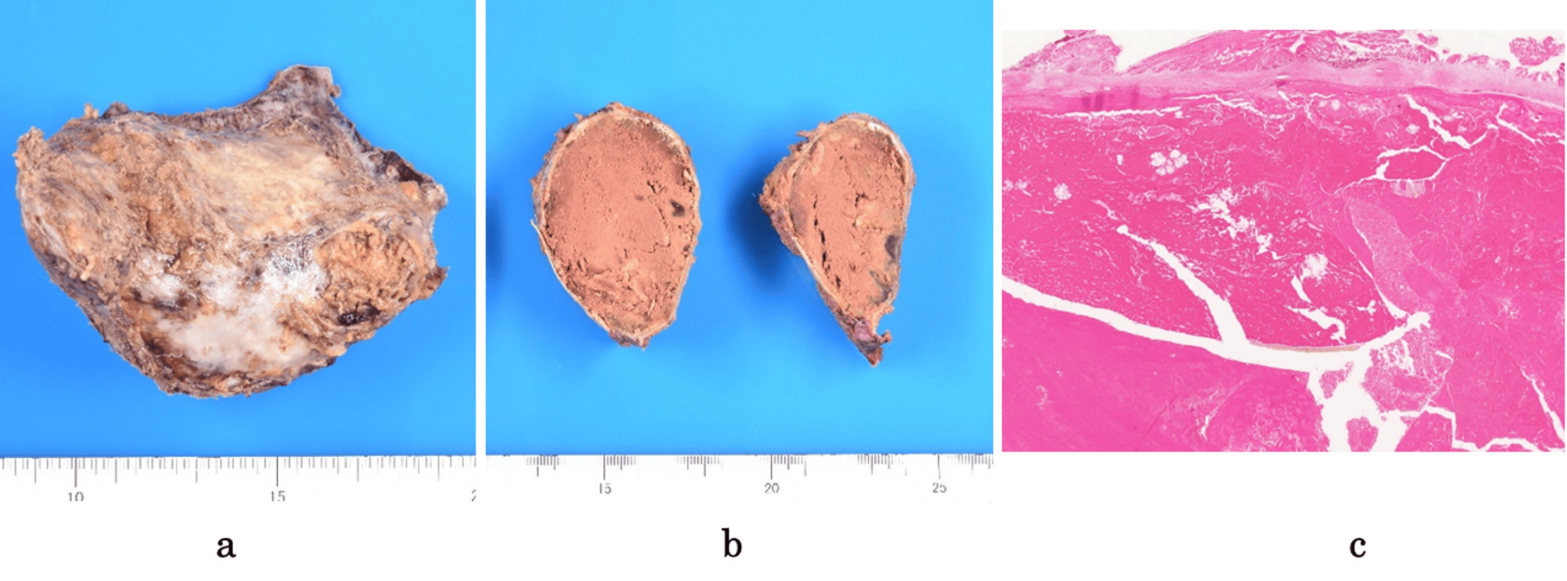
Resected specimen and pathological findings (a) Gross photograph of the resected mass demonstrating a well-circumscribed, calcified mass measuring 110 × 70 mm. (b) Cut section of the specimen showing a chronic hematoma filling the entire specimen. (c) Microscopic examination revealing a chronic hematoma and no evidence of malignancy.

The excised specimen measured 110 × 70 mm and was surrounded by a calcified capsule (Figure 4). Grossly, the lesion was filled with an old hematoma. Pathological examination revealed a fibrous capsule with calcification. The lesion was filled with an old hematoma that contained hemosiderin deposits, and no signs of malignancy were noted.

**Figure 5:**
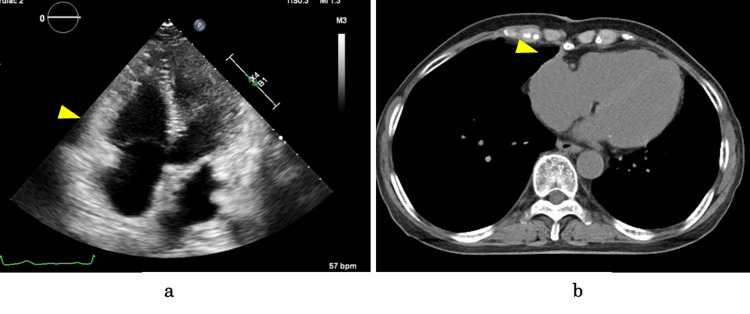
Postoperative echocardiography and computed tomography Postoperative echocardiography demonstrating (a) the resolution of cardiac compression (arrow) and improvement in the right ventricular function and (b) the absence of residual mass or recurrence (arrow).

The patient required intensive care for four days postoperatively. Postoperative echocardiography and CT verified the total resection of the mass and alleviation of cardiac compression (Figure 5). The hematoma did not recur during the one-year follow-up.

## Discussion

CEH is a rare condition characterized by a hematoma that slowly expands over several months to years following a traumatic event or surgery [1]. CEH has been reported to occur in various anatomical locations, including the extremities or intra-abdominal compartment [2,3]. However, the pathogenesis underlying the persistent expansion of the hematoma without resorption remains elusive; moreover, it is hypothesized to share similarities with chronic subdural hematoma [4]. The lesion's continuous growth appears to be influenced by inflammatory responses to the hematoma and is possibly caused by the irritating effects of blood and its breakdown products. These effects lead to bleeding from the capillaries within the granulation tissue.

CEH originating from the pericardium is a rare occurrence. According to the literature review, a total of 16 cases were published in English between 1990 and 2021 [5]. Most case reports have indicated that pericardial CEH often develops following blunt chest trauma or cardiac surgery [6-10]. In some cases, the underlying cause is coronary-to-pulmonary artery fistula or coronary aneurysm [11,12]. This condition also developed following minimally invasive procedures such as catheter ablation [13]. In the present case, the specific event that triggered the hematoma formation was not identified. Idiopathic CEH may be caused by the rupture of small vessels secondary to minor trauma or inflammation [14].

CEH has a variable growth rate, with Takahashi et al. reporting a mean interval of 5.8 (1.7-14) years between the precipitating event, such as trauma or surgery, and subsequent treatment of CEH [5]. Some studies have reported CEH cases diagnosed after 30-50 years [7-10]. The slow-expanding nature of CEH necessitates differential diagnosis from malignancy in idiopathic cases.

Most of the reported cases presented with heart failure symptoms secondary to cardiac dilation caused by compression by an expanding hematoma [5]. To relieve heart compression, symptomatic cases often necessitate hematoma resection. However, in the present case, the diagnosis was made incidentally on imaging because of the paucity of clinical symptoms. Although asymptomatic, this patient exhibited marked right-heart compression and mild right ventricular outflow tract stenosis. Progressive hematoma enlargement inevitably results in heart failure. Furthermore, in idiopathic CEH, tumor-induced bleeding should be considered a potential etiology, and hematoma resection may be indicated for diagnosis and treatment.

The structure is characterized by a central blood clot surrounded by granulation tissue and a peripheral layer of dense fibrous tissue [5,6,14]. In numerous cases reported, calcification was observed to extend beyond the hematoma capsule, often involving the underlying epicardium [8]. Chronic inflammation of the hematoma may propagate to the pericardium, thereby inducing secondary constrictive pericarditis. Apart from the calcification of the hematoma capsule in our patient, calcification was noted around the left atrial appendage. Although no pericardial findings other than adhesions were noted intraoperatively, chronic inflammation from the hematoma could have resulted in constrictive pericarditis.

CEH can be diagnosed using a combination of ultrasound, CT, and MRI. The lesion exhibited peripheral enhancement on contrast-enhanced CT. In addition, the characteristic MRI finding is a "mosaic sign" demonstrating a mixture of high- and low-signal intensities within the lesion on T2-weighted images [15]. In the present case, the CT images did not demonstrate enhancement but revealed scattered calcifications and heterogeneous attenuation within the lesion. The MRI findings did not exhibit the typical "mosaic sign" but showed a relatively homogeneous appearance. Imaging characteristics may vary depending on the degree of coagulation within the hematoma [16].

The surgical procedure commenced with an off-pump technique. However, intraoperative findings revealed extensive adhesions causing cardiac injury, necessitating conversion to on-pump cardiopulmonary bypass to ensure safe surgical dissection. Despite the successful completion of the on-pump procedure and successful gross total resection of the idiopathic CEH, the intraoperative findings of extensive adhesions secondary to the surrounding inflammatory response underscored the need for more aggressive preoperative planning in similar cases.

## Conclusions

Pericardial CEH, although rare, can lead to significant clinical complications, including cardiac compression and heart failure. In light of these potential risks, the appropriate surgical management is of critical importance. However, the surgical indication for CEH in asymptomatic patients remains unclear. Furthermore, given the necessity of differentiating between tumors and other potential etiologies, active surgical resection should be considered. This case highlights the importance of recognizing CEH as a differential diagnosis for mediastinal masses and the need for careful evaluation to determine the optimal management strategy.
